# Optimal distribution of VLBI transmitters in the Galileo space segment for frame ties

**DOI:** 10.1186/s40623-023-01926-0

**Published:** 2023-11-17

**Authors:** Helene Wolf, Johannes Böhm

**Affiliations:** Department of Geodesy and Geoinformation, TU Wien, 1040 Vienna, Austria

**Keywords:** VLBI, Galileo, VLBI transmitter, Frame ties

## Abstract

Equipping Galileo satellites with a VLBI transmitter (VT) will allow to observe satellites next to quasars with Very Long Baseline Interferometry (VLBI) radio telescopes. This concept will facilitate the direct estimation of the satellite orbits in the celestial reference frame. Moreover, these observations along with usual Galileo observations can be used to transfer the space tie between the VT and the antenna on the Galileo satellite to the Earth surface realizing the frame tie at the geodetic site with VLBI radio telescope and Galileo antenna. In this study, we assess the accuracy of that frame tie by simulating the estimation of station coordinates from VLBI observations to Galileo satellites next to quasars. We find that at least two or three satellites need to be equipped with a VT with the best results if all satellites with a VT are placed in the same plane. Concerning the ratio between satellite and quasar observations within a schedule, the results suggest that the optimal ratio is around 30% to 40% satellite observations out of the total number of observations in order to have enough observations for the estimation of the station coordinates but still enough quasar observations to ensure a sufficient sky-coverage for the estimation of troposphere parameters. The best scenario with two satellites yields repeatabilities for the east and north components between 7.5 and 10 mm, and for the up component between 9.5 and 12 mm. In case there is a third satellite with a VLBI transmitter in the same plane, the repeatabilities are reduced by up to 2 mm for the horizontal components and up to 3 to 4 mm for the up component. Rotating the schedules over the constellation repeat cycle of Galileo of 10 days reveals that there are differences between the individual days, but there are no days with a significantly worse precision of the estimated station coordinates.

**Graphical Abstract:**

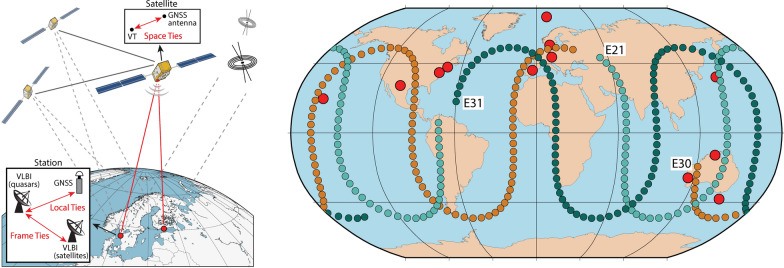

## Introduction

**Fig. 1:**
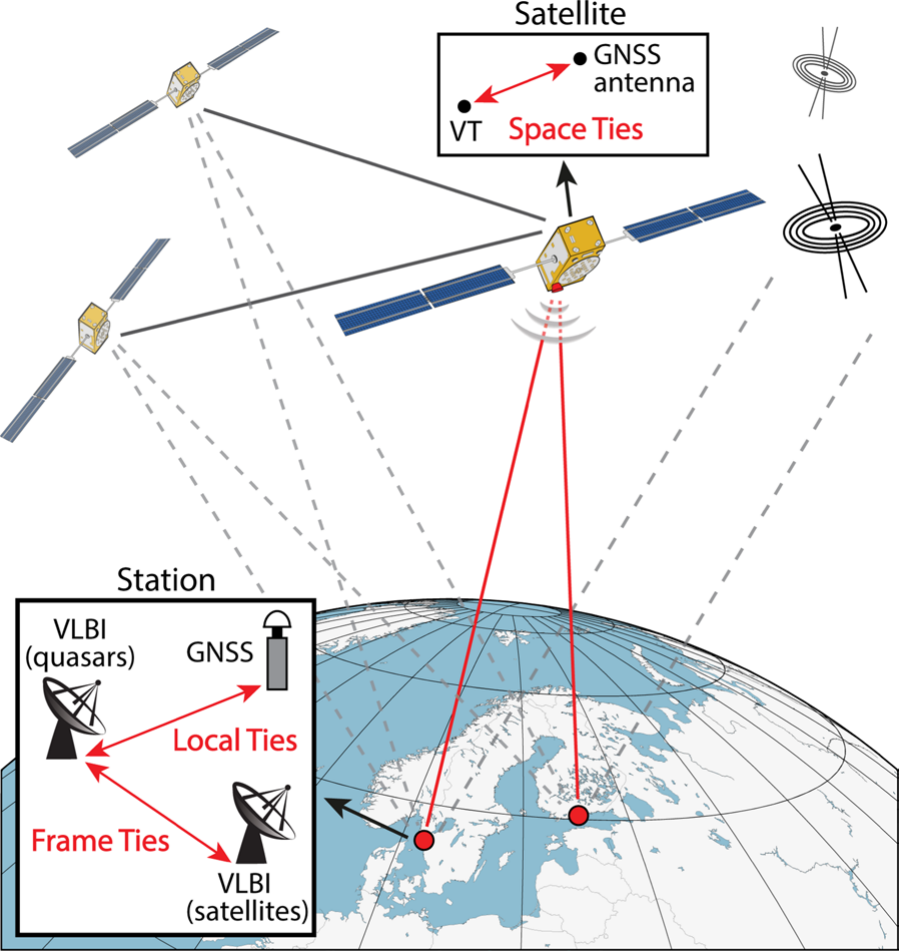
Illustration of a Galileo satellite observed with VLBI radio telescopes and GNSS antennas on the ground. This concept will allow the transfer of the space tie to the local tie at the Earth surface. Moreover, the station coordinates of the radio telescope can also be determined from observations to quasars, thereby realizing the frame tie

Very long baseline interferometry (VLBI) is a space geodetic technique observing the emission from extragalactic radio sources, mostly quasars, with a network of radio telescopes. The mounting of a VLBI transmitter (VT) on board of satellites will allow to observe satellites next to quasars with VLBI radio telescopes, opening new possibilities but also new challenges. Besides Satellite Laser Ranging (SLR), Doppler Orbitography by Radiopositioning Integrated by Satellite (DORIS), and Global Navigation Satellite Systems (GNSS), VLBI is one of the four space geodetic techniques contributing to the determination of the International Terrestrial Reference Frame (ITRF; Altamimi et al. 2023). The results from these four techniques are combined in order to overcome technique-specific weaknesses, making use of the strengths of the individual techniques as well as of local ties. These local ties represent vectors between geodetic instruments at co-location sites determined by local surveys. The comparison of the local tie vectors with results from space geodesy, however, shows significant discrepancies at the centimeter level (Altamimi et al. 2016), constituting one of the limiting factors for the accuracy of International Terrestrial Reference System (ITRS) realizations. In other words, the consistency between local ties and space geodetic estimates needs to be refined for further improvements of the ITRF (Boucher et al. 2015). This goal could be achieved by observing a satellite with VLBI and GNSS (Fig. 1) with well calibrated ties between the instruments on the satellite.

The co-location satellite Genesis, combining all four space geodetic techniques, with a polar orbit in about 6000 km altitude (Delva et al. 2023) is scheduled for launch in 2027/2028 by the European Space Agency (ESA). One issue with the planning of missions like Genesis is related to the assessment of the benefit of the space tie. Here, one option would be to determine and compare the positions of the technique-specific antennas on the satellite (space ties; Rothacher et al. 2009); however, this concept is subject to various complications including the availability of software to estimate orbits from all techniques (Klopotek et al. 2020). Along that path, Wolf et al. (2022) have assessed the orbit determination with VLBI observations based on dilution-of-precision (DOP) values. Investigations concerning the contribution of VLBI observations to satellites to the concept of co-location in space were carried out by Männel et al. (2016).

Alternatively, we can assume that the orbit of the co-location satellite is determined from the satellite techniques (GNSS, SLR, DORIS) alone. Then, we use VLBI observations to the satellite to derive station positions, i.e., we determine VLBI station coordinates in the dynamic frame realized with the satellite orbits. These coordinates are then compared against VLBI station coordinates from quasar observations, thereby providing information about the tie between the dynamic and the kinematic (quasar) frame. With the absence of real observations and based on simulations, we focus on the precision of station coordinates from VLBI observations to satellites to get an estimate of the accuracy of the frame tie. This concept was used by Plank et al. (2014) for different satellites and by Anderson et al. (2018) in their simulations with a Genesis-like satellite.

In the last years, real VLBI observations to satellites were carried out as first attempts. Haas et al. (2014, 2015) and Hellerschmied et al. (2014) describe observations carried out using the antennas Wettzell (Germany) and Onsala (Sweden) to GLONASS satellites with the goal to test the L-band capabilities of the antenna Wettzell.

Haas et al. (2017) investigated VLBI observations to GNSS satellites performed on an intercontinental baseline between Onsala (Sweden) and Hartebeesthoek (South Africa). Further, Plank et al. (2017) and Tornatore et al. (2014) successfully observed GNSS satellites with two VLBI telescopes forming a single baseline with the goal to evaluate the feasibility of VLBI telescopes observing GNSS satellites and using as many available procedures and programs as possible. Hellerschmied et al. (2018) performed several experiments observing the nano-satellite APOD, combining SLR, GNSS and VLBI on one platform, with the AuScope VLBI array and developed a procedure chain from observing to analyzing the data.

**Fig. 2:**
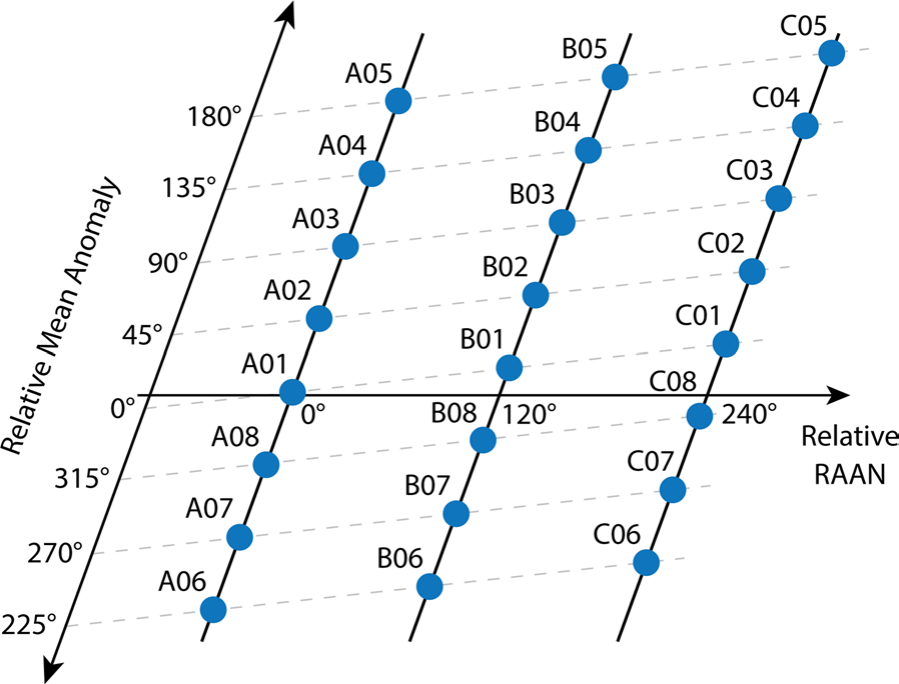
Orbital planes of the Galileo space segment with eight satellites per plane. The satellites are indicated by their slot assignments. The satellites are ordered by right ascension of the ascending node (RAAN) and the relative mean anomaly

Nowadays, no satellite mission with a dedicated VLBI transmitter (VT) is in operation, which could be observed with VLBI radio telescopes. However, besides Genesis, there are plans to mount a VT on board of Galileo satellites. Galileo is Europe’s Global Navigation Satellite System consisting of three orbital planes inclined at an angle of 56° to the equator and spaced by 120° to each other, see Fig. 2. Fully operational, it consists of eight operational satellites per plane at an altitude of 23,222 km.

Several studies already investigated VLBI observations to GNSS satellites with respect to the realization of frame ties between the VLBI and the satellite frame (Plank et al. 2016). Recently, frame ties with VLBI observations to GPS satellites in L-band were investigated by Schunck and McCallum (2023). However, from a practical point of view, there are outstanding questions, such as how many satellites should be equipped with a VLBI transmitter and how should those satellites be spread over the planes and among the slots within the planes. Further, the question arises what is the optimal ratio between the satellite and quasar observations within one schedule in order to optimize the results.

This study investigates the cases of equipping one, two or three satellites of the Galileo system with a VLBI transmitter. In total five different scenarios are considered regarding different distributions over the planes. For these five scenarios the repeatabilities of the station coordinates in the satellite frame are assessed, enabling the assessment of the precision of the tie between the satellite and the VLBI frame. In section Methods, we describe the settings of the scheduling and simulation of the VLBI observations. The section Results provides the findings of the different scenarios, and the last section with the Conclusions summarizes the work and gives recommendations with respect to the distribution of VTs on the satellites.

## Method

**Fig. 3:**
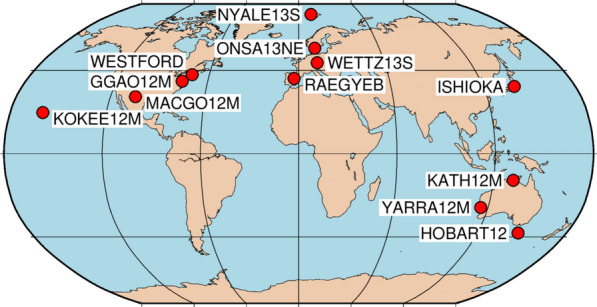
Network with 12 VGOS stations used in this study

Our study is based on a network of 12 VLBI Global Observing System (VGOS; Petrachenko et al. 2010) type stations (Fig. 3), and we use 24 h sessions starting on August 27, 2022 00:00:00 UTC. We investigate scenarios having either one, two or three satellites of the Galileo space segment equipped with a VT. The repeatabilities of the results for the scenario with one satellite are significantly higher compared to the results of the other scenarios and do not yield to usable precisions. Therefore, and as the inclusion of these results in the charts would degrade the readability of these, the results are not shown and only discussed. For the scenarios with two and three satellites equipped with a VT, this study examines various possibilities concerning the distribution of these satellites over the different planes A, B and C. In case three satellites are equipped with a VT, the different distributions among the planes, which are investigated, consist of firstly one per plane A, B and C, secondly three in plane A, and thirdly one in plane A and two in plane B. In case two satellites are equipped with a VT, two different scenarios are investigated, firstly two in plane A and secondly one per plane A and B.

Table 1 provides an overview of the five different scenarios with listing slot number and Space Vehicle Identifier (SV ID) of the satellites which are considered to be equipped with a VT. The selection of the different satellites within one scenario is performed in a way that the satellite ground tracks are not overlapping in order to ensure different observation geometries between the satellites and the observing stations. Wolf (2021) investigated the visibility of two and three Galileo satellites in the same or different planes. The selection of the satellites for the different scenarios in this study is done based on these outcomes in order to optimize not only the visibility, but also the occurrence of different observation geometries.

**Table 1:** Overview of the five scenarios and the considered satellites

Scenario	Satellites		
1 in A, B, C	A01 (E31)	B08 (E26)	C07 (E08)
3 in A	A01 (E31)	A03 (E21)	A05 (E30)
1 in A, 2 in B	A01 (E31)	B06 (E12)	B08 (E26)
2 in A	A01 (E31)	A05 (E30)	–
1 in A, B	A01 (E31)	B08 (E26)	–

**Fig. 4:**
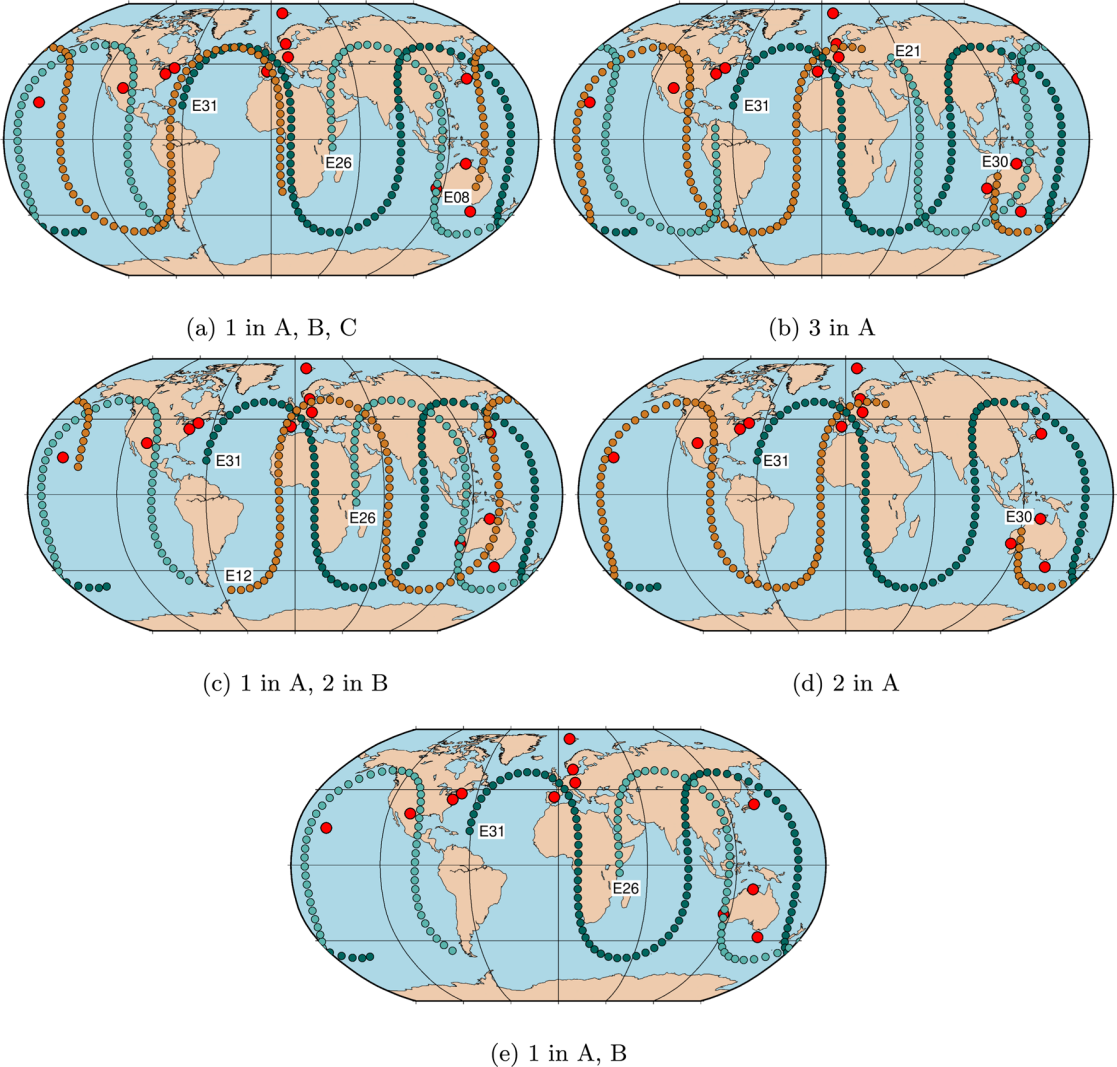
Ground tracks of the satellites considered in the different scenarios on the day of analysis (August 27, 2022). The dots represent the position of the satellite over Earth in a 15-min interval without considering the visibility of the satellite from the VLBI network

However, it is important to note, that the observation geometry between the satellites and the stations is always changing due to the rotation of the Earth. So even if the satellites are placed in the same plane in slots right after each other, the geometry will not be identical when the second satellite reaches the position of the first satellite. Figure 4 shows the satellite tracks of the different satellites for the five scenarios on August 27, 2022. For each scenario various schedules are created with different ratios between the quasar and satellite observations. Here, the ratios of 10% to 60% satellite observations of the total number of observations are investigated. For the scenario with one satellite in plane A and B, only the results for the ratios 10% to 40% are shown as the repeatabilities for 50% and 60% are much higher compared to the results of the other scenarios.

### Scheduling

For generating the schedules, the software VieSched++ is used (Schartner and Böhm 2019). This software has been equipped with a satellite scheduling module which enables to include satellite observations in a schedule together with quasar observations, either manually or automatically (Wolf 2021). For this study the satellite scans are scheduled in an automatic fashion, which means the optimal scan is chosen among all possible quasar and satellite scans. As the network consists of VGOS stations only, the scan length of both, satellite and quasar scans, is fixed to 10 s (Schunck and McCallum 2023). Further, in this simulation study the preob, field system and calibration times are set to 5 s in total. With that setting, we achieve a large number of short scans, which are very well distributed over the sky at the individual stations.

For each scenario different schedules are created with different ratios between the number of quasar and the number of satellite observations. This is realized by changing the weight of the satellite scans with respect to the weight of the quasar scans. In total, for each scenario six different schedules are created with 10%, 20%, 30%, 40%, 50% and 60% satellite observations of the total amount of observations.

### Simulation

The 24-h schedules are simulated 1000 times with the Vienna VLBI and Satellite Software (VieVS; Böhm et al. 2018). These Monte Carlo simulations are carried out by using three main error sources: tropospheric turbulence, clock errors, and the thermal noise. A tropospheric refractive index structure constant *C*_n_ of 1.8 × 10^−7^ m^−1/3^ with a scale height of 2000 m is assumed at all stations. The wind speed in the eastern direction is set to 8 m/s and no wind speed in the northern direction is simulated. The instability of the clock is described as the sum of a random walk and an integrated random walk process assuming an Allan Standard Deviation of 1 × 10^−14^ after 50 min. The measurement error is simulated for both observation types, quasar and satellite observations, as white noise with 10 ps as standard deviation. We want to emphasize here that we do neither simulate errors in the satellite orbits nor in the space tie on the satellite.

### Analysis

The analysis is performed based on the least-squares adjustment of the individual 24-h sessions. In the analysis, all five Earth Orientation Parameters (EOPs) as well as the coordinates of the quasars and the satellite orbits are fixed. We are using sp3 files from the International GNSS Service (IGS; Johnston et al. 2017) for the Galileo orbits, and we do not model the vector between the VT and the center-of-mass of the satellite. Since our analyses are based on simulations, the exact values of the a priori parameters are of no importance.

The station clocks are estimated with a quadratic term and piecewise linear offsets (PWLOs) every 60 min with a relative constraint of 1.3 cm between these offsets. This is done for all stations except for the station with the highest number of observations, which is selected as reference clock. The troposphere parameters are estimated from both observation types, namely satellite and quasar observations. The zenith wet delays are estimated every 10 min as PWLOs with a relative constraint of 1.5 cm and the northern and eastern gradients are estimated every 20 min as PWLOs with a relative constraint of 0.05 cm. Earlier studies have shown, that the large number of scans with VGOS observations allows short estimation intervals of troposphere parameters (Haas et al. 2023).

The station coordinates are estimated for all VLBI radio telescopes using satellite observations only in order to derive the station coordinates in the satellite frame. Observations to quasars are only used to estimate troposphere and clock parameters, i.e., the respective station coordinates in the quasar frame are fixed. No datum constraints are necessary as the rank defect of estimating station coordinates and fixing EOPs, geocenter and orbits using a satellite-quasar schedule is zero (Klopotek 2020). More precisely, no-net-rotation conditions (NNR) are not applied as EOPs are not estimated. Also, as the satellite observations are sensitive towards the origin, no-net-translation conditions (NNT) are not applied either. The reason for not applying any datum constraints is the intention to perform a least-squares adjustment with minimum constraints in order to avoid the mitigation of effects and a distortion of the network by over-constraining.

The estimates of the 1000 simulations are used to determine repeatability values for the station coordinates in the satellite frame, thereby assessing the frame tie with respect to long-term VLBI coordinates from quasar observations, which are assumed error-free. The station coordinates are first converted from the XYZ-frame to local east, north, and up (ENU) directions, and further the repeatability is determined as standard deviation of the estimated station coordinates. The different scenarios and schedules with various ratios between satellite and quasar observations are compared and assessed based on these repeatabilities.

## Results

### Numbers of scans and observations

**Fig. 5:**
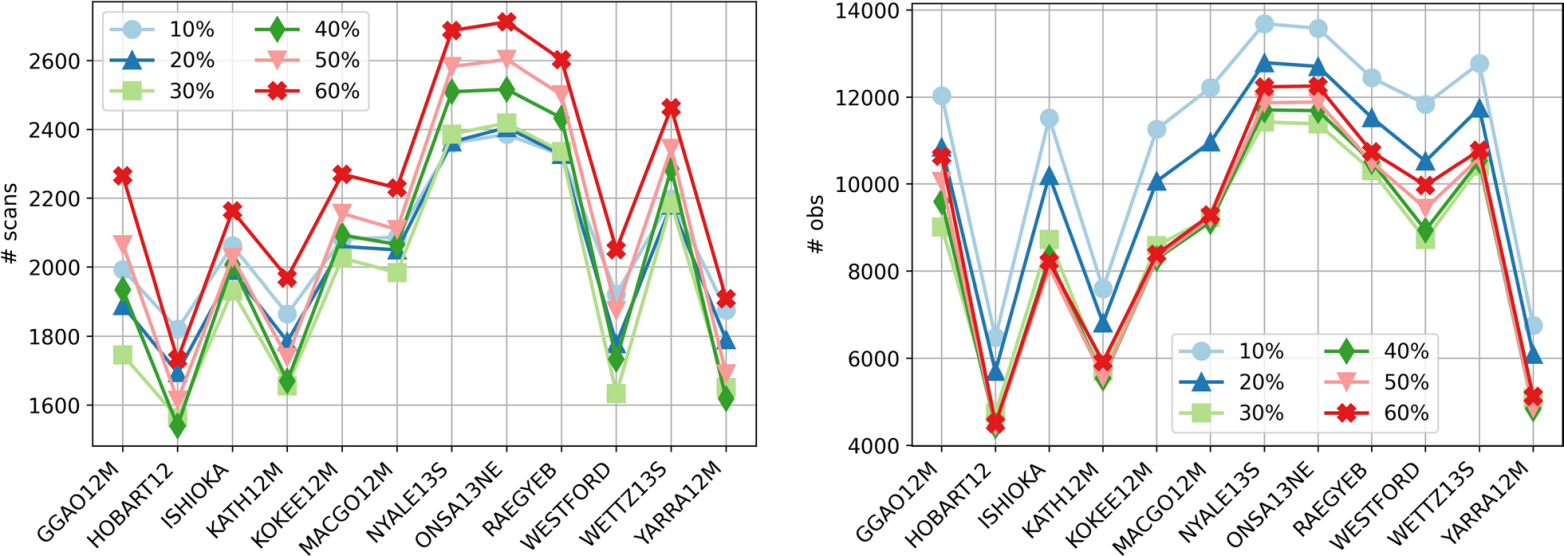
Total number of scans (left) and observations (right) for different ratios of satellite observations for the scenario having three satellites in plane A equipped with a VT

In total 30 schedules are created for the selected VGOS network considering different satellites and ratios between satellite and quasar observations. As an example, Fig. 5 shows the total number of scans and the total number of observations (satellite and quasar observations) of the individual stations for the different ratios for the scenario considering three satellites in plane A. It is evident that the stations in Europe have the largest number of scans and observations and the stations in Australia and WESTFORD have a significantly lower number of scans and observations. This is due to the distribution of the individual stations and different antenna specifications. The stations WESTFORD, GGAO12M, HOBART12, KATH12M and YARRA12M have significantly slower slew rates than the other stations. Therefore, these stations spend more time on slewing and reach a lower number of scans and observations. It is also clearly visible that for the schedules with a higher ratio of satellite observations all stations have a higher number of scans but a lower number of observations. This is due to the fact that smaller subnetworks are formed in order to observe the satellite with all the stations where the satellite is visible. This results in a higher number of scans but in a lower number of observations because there are less stations in the scans.

**Fig. 6:**
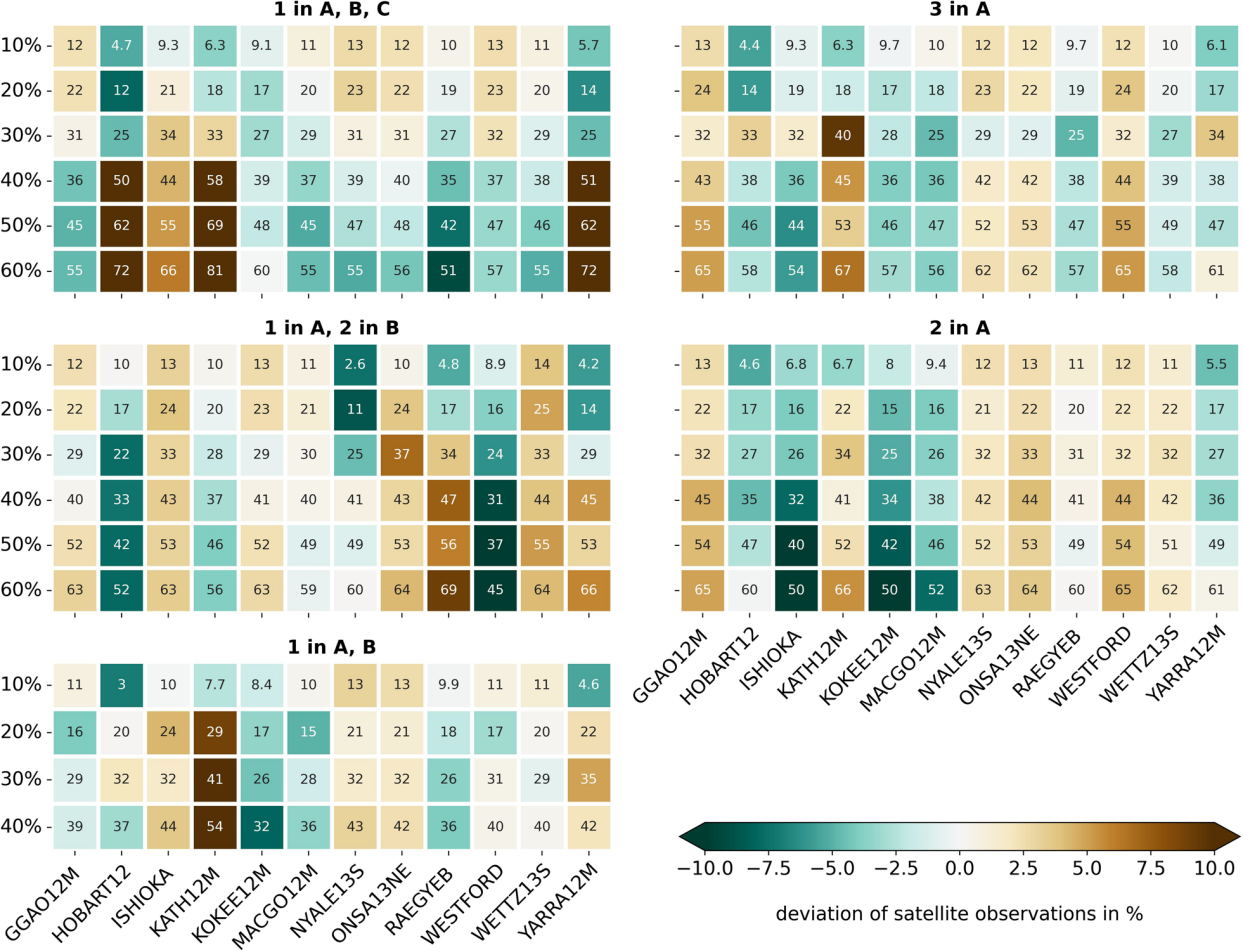
Deviation in % for the individual stations from the predefined ratio between satellite and quasar observations of the whole schedule, depicted as colors. The ratios are provided as numbers in the cells

The ratios of 10% to 60% satellite observations out of the total number of observations are fulfilled for the total number of satellite observations at all stations. It does not imply that for all individual stations the ratio between satellite and quasar observations is exactly fulfilled, for instance 10%. The colors in Fig. 6 depict the deviation in % of the percentage of the number of satellite observations for the individual stations. It is evident that higher ratios of satellite observations cannot always be achieved for every station due to a limited visibility of the satellite. For these schedules, some stations have a higher ratio of satellite observations while some other stations have a lower ratio. As an example, in the scenario considering one satellite in A and two satellites in B for a ratio of 60% satellite observations, the ratio for the station RAEGYEB is 69% but for the station WESTFORD only 45%.

### Sky-coverage

**Fig. 7:**
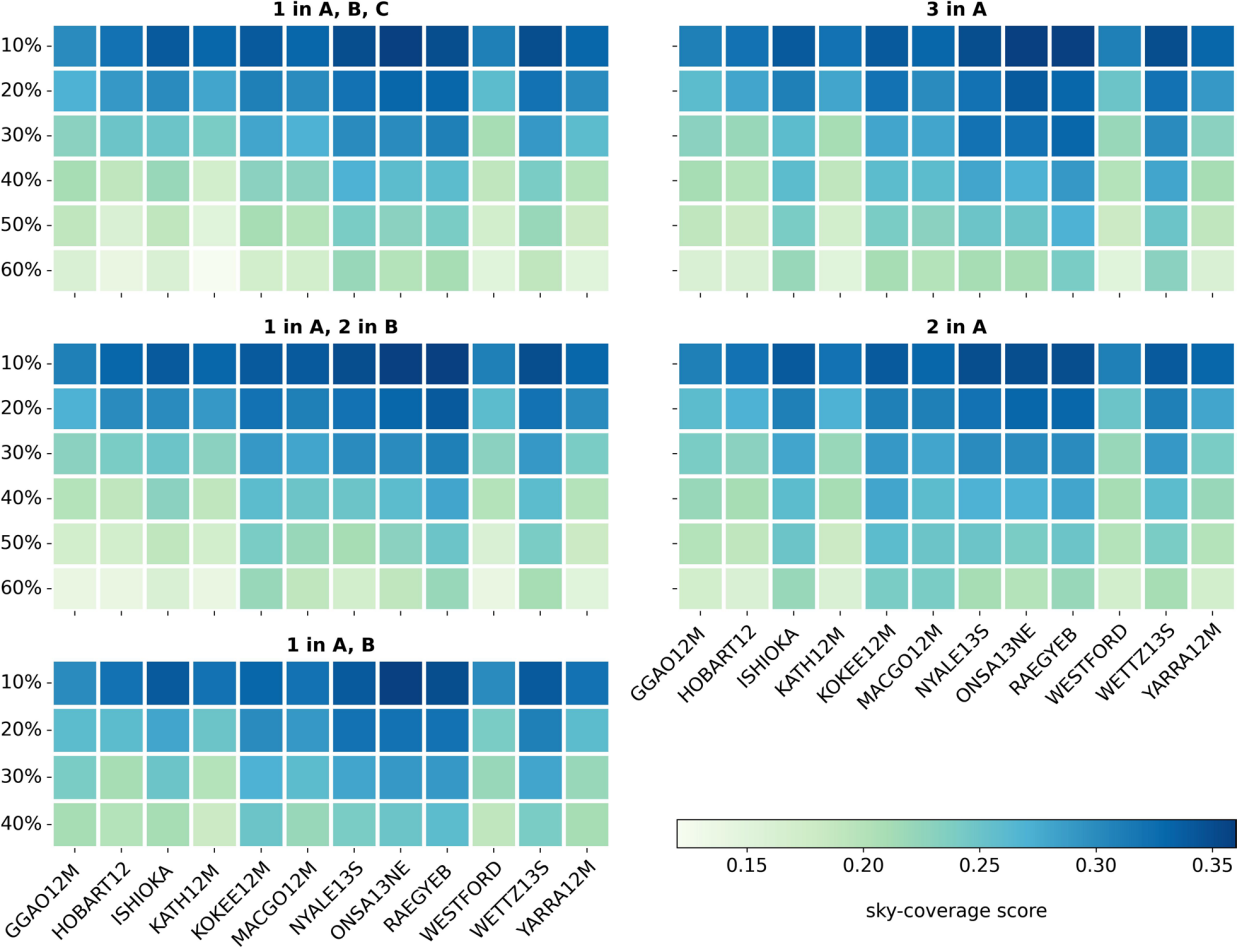
Sky-coverage score for 37 areas after 8 min. The higher the value the better the distribution of the scans on the sky above a station

A good distribution of the scans over the sky above the stations is very important for the determination of the troposphere parameters and for the estimation of the station coordinates. The sky-coverage can be assessed with a so-called sky-coverage score. This score is calculated in VieSched++ by splitting the sky above a station into smaller areas of approximately equal size, analyzing which areas are covered by at least one scan, and dividing the covered areas by the total number of areas. The sky-coverage score ranges from zero to one, with one denoting a perfect sky-coverage, meaning that every area is covered with at least one scan (Schartner and Böhm 2019). Figure 7 represents the sky-coverage score based on 37 areas with an 8-min time interval for the different scenarios and ratios, which is the most demanding setting possible in VieSched++.

It is clearly visible that for all scenarios the sky-coverage score becomes smaller representing a worse distribution of scans over the sky with an increasing percentage of satellite observations in a schedule. Due to the reason that satellite observations can be carried out either to three or two satellites only, the distribution of these observations over the sky at a station is limited. A higher percentage of satellite observations in a schedule thus means more observations to satellites, which cannot be well distributed. Therefore, the sky-coverage is degrading with more satellite observations.

A comparison of the different scenarios in terms of sky-coverage shows that for a ratio of 10% the results are similar. However, with increasing the ratio of satellite observations, the scenarios with one satellite per plane A, B, C and one satellite in A and two satellites in B have smaller values than the scenario with three satellites in plane A. This difference can be related to the ground tracks of these three scenarios; see Fig. 4. The ground tracks of the satellites for the scenario with three satellites in plane A are neither partly overlapping as it is the case in Fig. 4a nor are they lacking coverage in the South Pacific as it is the case for the scenario with one satellite in A and two satellites in B see Fig. 4c.

In Fig. 7 it can be recognized that the stations HOBART, KATH12M, and YARRA12M in Australia, but also two stations in Northern America, namely WESTFORD and GGAO12M, have a worse sky-coverage score than the other stations. This holds for every scenario and for all ratios, because these stations have significantly less scans compared to the other stations; see Fig. 5. This is due to the above-mentioned slower slew rates of WESTFORD, GGAO12M, HOBART12, KATH12M and YARRA12M compared to the other stations. Therefore, these stations reach a worse sky-coverage as they spend more time on slewing than faster VGOS stations.

### Repeatability of station coordinates

**Fig. 8:**
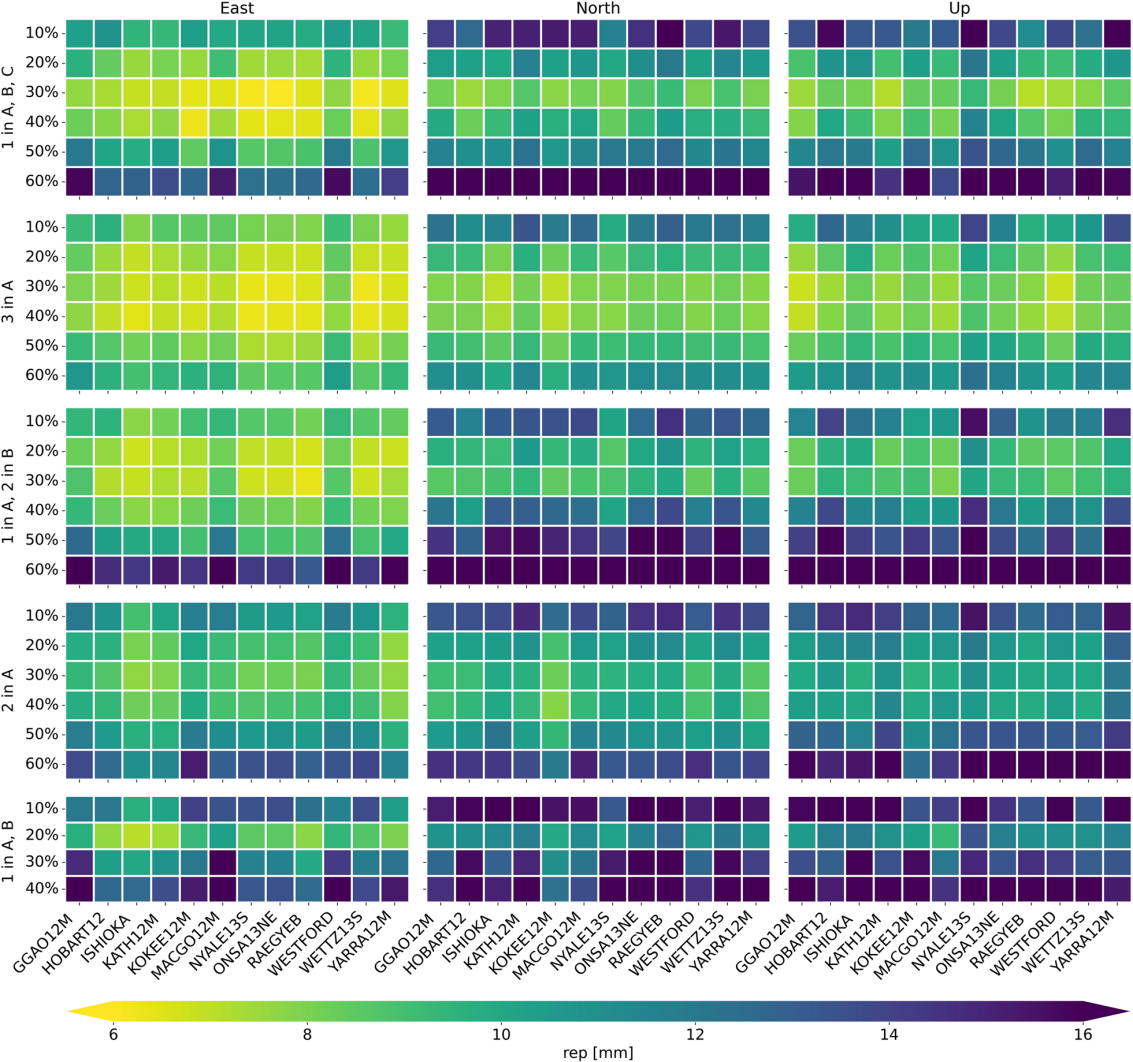
Station coordinate repeatabilities for the east, north, and up component for the simulations of all five scenarios. The results for having only one satellite equipped with a VT and the ratios of 50% and 60% satellite observations for the scenario with one satellite in plane A and B are not shown here as the repeatabilities are with 30 mm and far above much higher compared to the other results

Figure 8 depicts the repeatability of the station coordinates for the three components east, north, and up for the different scenarios and percentages of satellite observations. It is evident that the scenario with three satellites in plane A yields the best results for all different ratios of satellite and quasar observations. In particular, the lowest repeatabilities are achieved for ratios of 30% and 40% satellite observations with values between 6.3 and 8 mm for the east component, 6.9 and 8.4 mm for the north component, and between 6.8 and 8.8 mm for the up component. A ratio of 30% satellite observations is also the best approach for the other scenarios with three satellites. This is due to the fact that ratios of 10% and 20% result in too few observations for the estimation of the station coordinates, as only satellite observations are used. Ratios of 50% and higher result in fewer quasar observations and more satellite observations, leading to the problem of not well distributed observations over the sky above the stations as described in the previous section. The degrading sky-coverage results in worse estimates of troposphere parameters, which further has a negative impact on the precision of the estimated station coordinates.

The different performances of the three scenarios with three satellites in terms of the precision of the estimated station coordinates can be explained by the ground tracks of the satellites over 24 h, see Fig. 4. When comparing the ground tracks in Fig. 4a–c it can be seen that in (a) the ground tracks are partly overlapping and in general just shifted a little in longitude. Therefore, the observation geometries between observing baselines and the satellites are similar. The latter problem also applies to (c). In case (b) the three different satellites have varying trajectories and therefore different observation geometries occur, which lead to a higher precision in the estimated station coordinates.

**Fig. 9:**
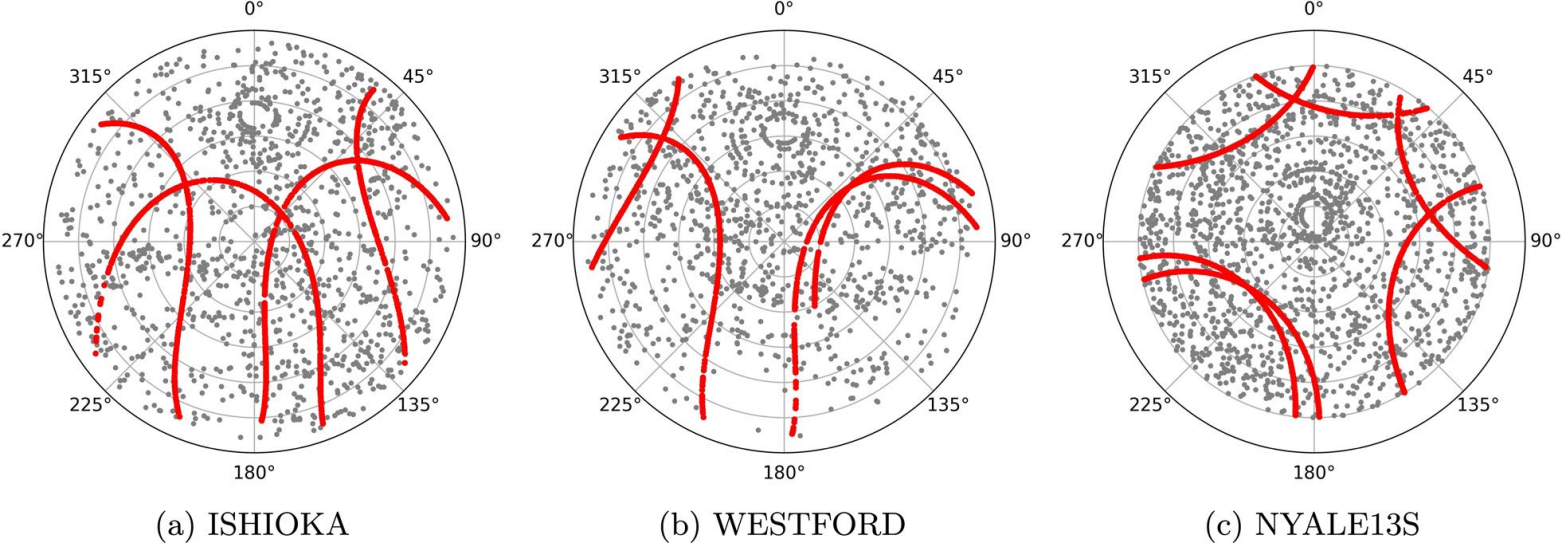
Distribution of the satellite and quasar observations on local skies for three stations based on the 24 h schedules on August 27, 2022 for the scenario with one satellite per plane** A**,** B**,** C** with a ratio of 30% satellite observations

The comparison of the results for the different components shows that the precision of the east component is better compared to the precision of the north and up component. We attribute this feature to the mostly north–south oriented ground tracks of the satellites. For stations at low latitudes, the up component can be estimated more precisely than the north component, due to the so-called north hole of the Galileo space segment caused by an inclination of 56° to the equator. This phenomenon is evident from the skyplots for the stations ISHIOKA and WESTFORD in Fig. 9a, b. Both stations have a sufficient number of satellite observations in the eastern, western, and southern part of the sky, but no observations in the northern part. For the station NYALE13S the situation is different, as this station is located far north. Therefore this station has no observations in the zenith direction and the up component is determined worse compared to the other stations; see Fig. 9c. Further, the results for scenarios with three satellites and the east component suggest that the stations WESTFORD, MACGO12M, and GGAO12M have higher repeatabilities at all ratios compared to the other stations. This can be explained by the smaller number of scans and observations and therefore worse sky-coverage for these stations. However, it is not yet fully understood, why this effect is not as clear in the north and up component.

The best result for the scenario with two satellites in plane A is achieved for a ratio of 30% and for the scenario with one satellite per plane A and B for a ratio of 20%. This is due to the smaller number of satellites and similar ground tracks of these two satellites, leading to a worse sky-coverage for higher ratios of satellite observations. The reason for only displaying results for ratios up to 40% for the scenario with one satellite in plane A and one in B is that the repeatabilities for higher ratios are much higher (30 mm and far above; Fig. 8).

The repeatabilities of the station coordinates for the scenario with one satellite per plane A and B are significantly worse compared to the results of the scenario with two satellites in A. This is due to the very similar observation geometries between the stations and the satellites as the trajectories of these satellites are very similar and just shifted in longitude for one satellite in plane A and B; see Fig. 4d. For the scenario with two satellites in plane A the trajectories of the two satellites differ and therefore various observation geometries occur which result in a higher precision of the estimated station coordinates. The best result shows repeatabilities between 7.7 and 9.4 mm for the east component, 8.2 and 10 mm for the north component, and between 9.5 and 12 mm for the up component.

The results of the scenario with one satellite in plane A are not shown here as these do not yield to sufficiently good results. However, for this scenario three schedules are investigated with a ratio of 20%, 30% and 40% of satellite observations. A ratio of 20% yields to the best results with repeatabilities between 23 and 56 mm for the east component, 29 and 61 mm for the north component, and 38 and 68 mm for the up component. If the ratio of satellite observations is increased to 40% the repeatabilities are between 79 and 318 mm for the east component, 115 and 304 mm for the north component, and 130 and 270 mm for the up component.

### Changes over constellation repeat cycle

The Galileo space segment has a repeat cycle of 10 days. Within this period the constellation of the satellites and therefore the visibilities and observation geometries are changing. After these 10 days, the system constellation repeats again. Therefore, an investigation of the precision of the station coordinates over this cycle is of major importance.

**Fig. 10:**
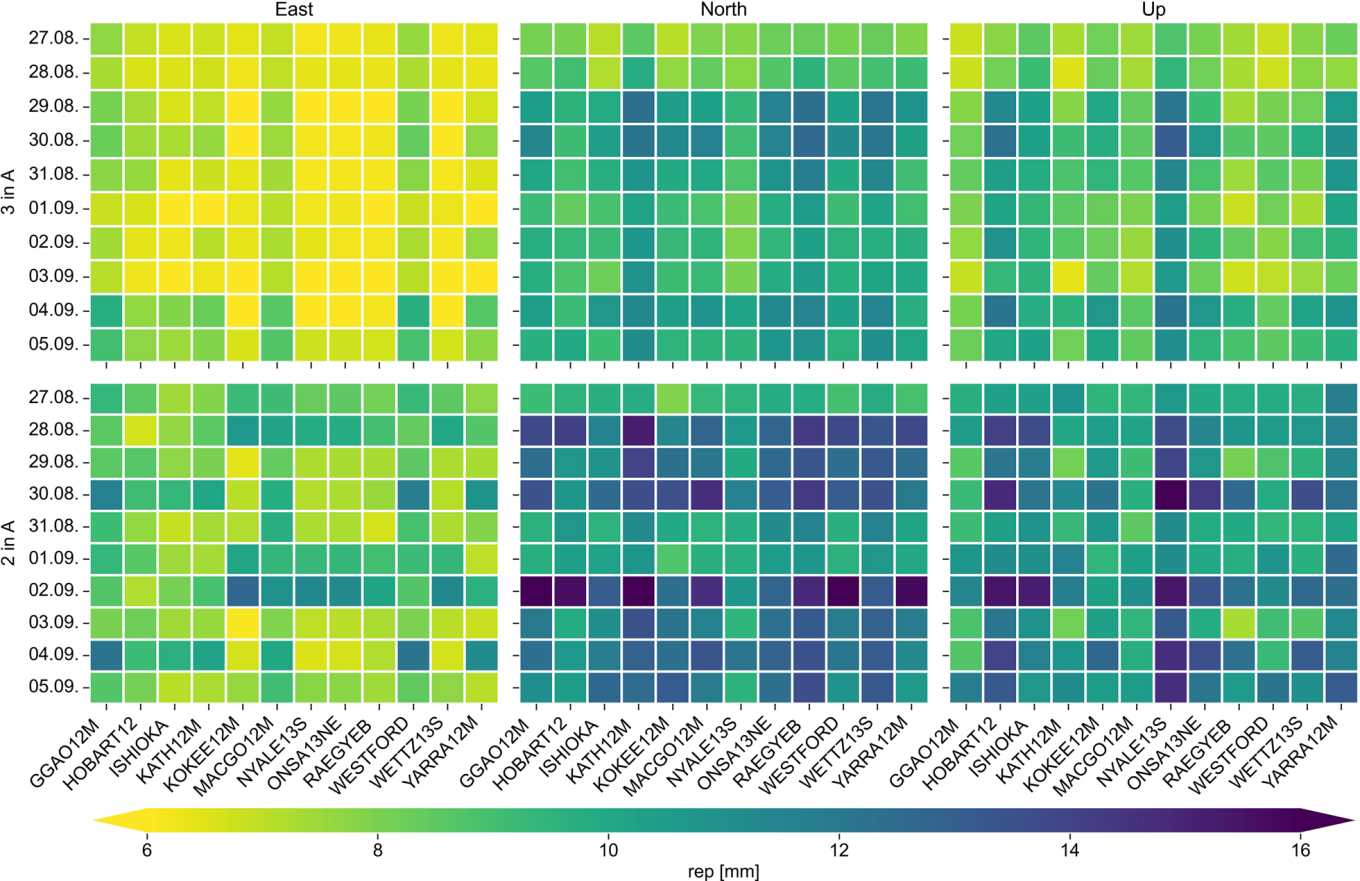
Station coordinate repeatabilities for the east, north, and up components for the scenarios with three satellites in plane A with a ratio of 40% and two satellites in plane A with a ratio of 30% of satellite observations over the 10-day repeat cycle of Galileo

For both cases, two and three satellites being equipped with a VT, the best scenarios, which are two satellites in plane A with a ratio of 30% and three satellites in plane A with a ratio of 40% are analyzed over the repeat cycle of Galileo, namely 10 days starting on August 27, 2022. The station coordinate repeatabilities for the east, north, and up components for both scenarios over the repeat period are shown in Fig. 10.

The precision of the estimated station coordinates varies over the days due to the aforementioned change of the ground tracks of the satellites resulting in different visibilities and observation geometries. For the scenario with three satellites and considering all three components, the overall results are best for the first 2 days. Over the remaining days the precision is worse for the north and up component, while for the east component there are days when the precision is even better. However, there is not 1 day with significantly worse results. The scenario with two satellites achieves the best precision for all components on August 31, 2022. The day with the worst precision is September 2, 2022. On that day, especially the north component is affected at some stations. By comparing the results of three and two satellites in plane A over the repeat period of Galileo, it is clearly visible that the scenario with three satellites performs significantly better, which was to be expected. For the scenario with only two satellites in plane A, the performance on most of the days is significantly worse compared to a few days when all components are determined with a precision better than 13 mm.

### Adding VT on further Galileo satellites

The results for two satellites in the same plane are less precise than the results for three satellites in the same plane. Although two satellites being equipped with a VT are already sufficient to derive station coordinates in the satellite frame, three satellites will lead to a higher precision. Further, four satellites in the same plane lead to a small improvement of the station coordinate repeatabilities by about 2 mm compared to three satellites. With in total five satellites, station coordinate repeatabilities improve by 0.5 mm compared to four satellites. However, having the fifth satellite in the same plane does not lead to an improvement. There is no gain in precision with every additional satellite.

### Future VGOS networks

The network used in this study (see Fig. 3) consists of currently available VGOS stations. Since future VGOS networks will be extended with new VGOS stations, the precision of estimated station coordinates is investigated accordingly. Three fictive VGOS stations are assumed to be placed at the sites of AGGO, BADARY, and HART15M having the same specifications as WETTZ13N, i.e., 13.2 ms diameter and slew rates of 12°/s and 6°/s in azimuth and elevation, respectively. These three additional stations increase the visibility of the satellites enormously, as these stations extend the network globally with positions in South America, South Africa, and China. Consequently, observation geometries and the precision of station coordinates are improved.

The performance of the scenario with three satellites in plane A with a ratio of 30% satellite observations is investigated using this 15 station network. The precision of the station coordinates is improved for all stations and for all three components east, north, and up. The repeatabilities are between 3.5 and 5.6 mm, which represents an improvement between 2 and 5 mm for the individual stations and components compared to the results of the same scenario using the 12 station network. These results show, that a globally extended network has the potential to further improve the estimation of station coordinates from satellite observations.

## Conclusions and outlook

This study examines the estimation of station coordinates from VLBI observations to Galileo satellites next to quasars. By determining the station coordinates from satellite observations alone, we assess the frame tie between the satellite and the quasar frame at the level of station coordinates. This approach provides information how well space ties from the Galileo satellites can be transferred to the Earth surface. In particular, we address three specifications, i.e., the number of Galileo satellites which have to be equipped with a VT, their distribution over the planes, and the ratio between satellite and quasar observations within a schedule.

The simulation results show that equipping one satellite with a VT is not enough, at least if it is observed over 24 h only. The station coordinate repeatabilities from 24 h sessions are above 30 mm. An interesting finding is that the optimal placing of two or three satellites with a VT would be within the same plane. Having two satellites with a VT in the same plane leads to better results than having three satellites with a VT in different planes. This shows that a higher number of satellites does not necessarily mean better results, but a proper placing of the satellites is more important. Future studies will also deal with the stacking of solutions from 24 h sessions addressing the question, how many weeks of satellite observations do we need to achieve frame ties at the Earth surface at the 1 mm level.

In future studies, we will also address the influence of errors in the space ties and other parameters on our estimates. Moreover, we will investigate the simultaneous estimation of the right ascension of the ascending node of the satellite orbits and of Earth orientation parameters and its impact on the solutions.

This study also clearly demonstrates that quasar scans are very important for the determination of the troposphere parameters and therefore for the estimation of station coordinates. A schedule with 100% satellite observations does not yield the best results for the estimation of station coordinates as the troposphere is dependent on a good sky coverage which is only guaranteed by including quasar observations in a schedule. If the individual stations do not achieve a good sky coverage caused by many satellite observations to the same satellites, the results of the station coordinates are degrading. In this study ratios of around 30–40% satellite observations of the total number of observations lead to the best results.

## Data Availability

The datasets used and/or analyzed during the current study are available from the corresponding author on reasonable request.

## Abbreviations

DOP: Dilution-of-precision
DORIS: Doppler Orbitography by Radiopositioning Integrated by Satellite
ENU: East, North, Up
EOP: Earth Orientation Parameters
ESA: European Space Agency
GNSS: Global Navigation Satellite System
GPS: Global Positioning System
IGS: International GNSS Service
ITRF: International Terrestrial Reference Frame
ITRS: International Terrestrial Reference System
NNR: No-net-rotation
NNT: No-net-translation
PWLO: Piecewise linear offset
RAAN: Right ascension of ascending node
SV ID: Space Vehicle Identifier
VGOS: VLBI Global Observing System
VieVS: Vienna VLBI and Satellite Software
VLBI: Very Long Baseline Interferometry
VT: Very Long Baseline Interferometry Transmitter
SLR: Satellite laser ranging
